# Moving from assessments to implementation: promising practices for strengthening multisectoral antimicrobial resistance containment capacity

**DOI:** 10.1186/s42522-023-00081-6

**Published:** 2023-04-14

**Authors:** Mohan P. Joshi, Fozo Alombah, Niranjan Konduri, Antoine Ndiaye, Ndinda Kusu, Reuben Kiggundu, Edgar Peter Lusaya, Robert Tuala Tuala, Martha Embrey, Tamara Hafner, Ousmane Traore, Mame Mbaye, Babatunde Akinola, Denylson Namburete, Alphonse Acho, Yacouba Hema, Workineh Getahun, Md Abu Sayem, Emmanuel Nfor

**Affiliations:** 1 USAID Medicines, Technologies, and Pharmaceutical Services (MTaPS) Program, Management Sciences for Health, Arlington, VA USA; 2 USAID Medicines, Technologies, and Pharmaceutical Services (MTaPS) Program, Management Sciences for Health, Abidjan, Côte d’Ivoire; 3 USAID Medicines, Technologies, and Pharmaceutical Services (MTaPS) Program, Management Sciences for Health, Nairobi, Kenya; 4 USAID Medicines, Technologies, and Pharmaceutical Services (MTaPS) Program, Management Sciences for Health, Kampala, Uganda; 5 USAID Medicines, Technologies, and Pharmaceutical Services (MTaPS) Program, Management Sciences for Health, Dar Es Salaam, Tanzania; 6 USAID Medicines, Technologies, and Pharmaceutical Services (MTaPS) Program, Management Sciences for Health, Kinshasa, Democratic Republic of the Congo; 7 Management Sciences for Health, Arlington, VA USA; 8 USAID Medicines, Technologies, and Pharmaceutical Services (MTaPS) Program, Management Sciences for Health, Bamako, Mali; 9 USAID Medicines, Technologies, and Pharmaceutical Services (MTaPS) Program, Management Sciences for Health, Dakar, Senegal; 10 USAID Medicines, Technologies, and Pharmaceutical Services (MTaPS) Program, Management Sciences for Health, Abuja, Nigeria; 11 USAID Medicines, Technologies, and Pharmaceutical Services (MTaPS) Program, Management Sciences for Health, Maputo, Mozambique; 12 USAID Medicines, Technologies, and Pharmaceutical Services (MTaPS) Program, Management Sciences for Health, Yaoundé, Cameroon; 13 USAID Medicines, Technologies, and Pharmaceutical Services (MTaPS) Program, Management Sciences for Health, Ouagadougou, Burkina Faso; 14 USAID Medicines, Technologies, and Pharmaceutical Services (MTaPS) Program, Management Sciences for Health, Addis Ababa, Ethiopia; 15 USAID Medicines, Technologies, and Pharmaceutical Services (MTaPS) Program, Management Sciences for Health, Dhaka, Bangladesh

**Keywords:** Antimicrobial resistance, AMR, Global Health Security Agenda, International Health Regulations, Joint External Evaluation, WHO benchmarks, Infection prevention and control, Antimicrobial stewardship, Multisectoral coordination, One Health

## Abstract

**Background:**

Antimicrobial resistance (AMR) poses a global threat to human, animal, and environmental health. AMR is a technical area in the Global Health Security Agenda initiative which uses the Joint External Evaluation tool to evaluate national AMR containment capacity. This paper describes four promising practices for strengthening national antimicrobial resistance containment capacity based on the experiences of the US Agency for International Development’s Medicines, Technologies, and Pharmaceutical Services Program work with 13 countries to implement their national action plans on AMR in the areas of multisectoral coordination, infection prevention and control, and antimicrobial stewardship.

**Methods:**

We use the World Health Organization (WHO) Benchmarks on International Health Regulations Capacities (2019) to guide national, subnational, and facility actions that advance Joint External Evaluation capacity levels from 1 (no capacity) to 5 (sustainable capacity). Our technical approach is based on scoping visits, baseline Joint External Evaluation scores, benchmarks tool guidance, and country resources and priorities.

**Results:**

We gleaned four promising practices to achieve AMR containment objectives: (1) *implement appropriate actions using the WHO benchmarks* tool, which prioritizes actions, making it easier for countries to incrementally increase their Joint External Evaluation capacity from level 1 to 5; (2) *integrate AMR into national and global agendas.* Ongoing agendas and programs at international, regional, and national levels provide opportunities to mainstream and interlink AMR containment efforts; (3) *improve governance through multisectoral coordination on AMR.* Strengthening multisectoral bodies’ and their technical working groups’ governance improved functioning, which led to better engagement with animal/agricultural sectors and a more coordinated COVID-19 pandemic response; and (4) *mobilize and diversify funding for AMR containment.* Long-term funding from diversified funding streams is vital for advancing and sustaining countries’ Joint External Evaluation capacities.

**Conclusions:**

The Global Health Security Agenda work has provided practical support to countries to frame and conduct AMR containment actions in terms of pandemic preparedness and health security. The WHO benchmarks tool that Global Health Security Agenda uses serves as a standardized organizing framework to prioritize capacity-appropriate AMR containment actions and transfer skills to help operationalize national action plans on AMR.

## Background

Antimicrobial resistance (AMR) continues to pose a global threat to humans, animals, and the environment, with dire consequences for the global economy and health security if it remains unchecked [1]. The most recent modeling estimated that 4.95 million deaths were associated with bacterial AMR in 2019, with western sub-Saharan Africa hit the hardest [2]. Although AMR is a worldwide problem, low- and middle-income countries (LMICs) carry a higher AMR burden [3], including deaths [2]. However, finding recent AMR data from individual LMICs is difficult. In a 2017 review of 54 African countries, 42.6% did not have acceptable published data on AMR [4], though other reviews of quality studies in Africa have shown that overall, clinical isolates were highly resistant to antimicrobial drugs [5], and specifically, that *E. coli* isolates had a high resistance percentage for recommended first- and second-line antibiotics [6].

Extended-spectrum β-lactamase (ESBL) production is a major mechanism for multidrug resistance in *E. coli* [7]. Table 1 shows a brief snapshot of the range of ESBL-positive *E. coli* isolates from various human, animal, and environmental sources in the 13 target countries described in this paper (12 in Africa and 1 in Asia—see Table 2).

**Table 1:** Prevalence of ESBL production in *E. coli* isolates from 13 target country studies

Country	ESBL-producing *E. coli* prevalence	Sample number/type	Reference
Bangladesh	67.5%, 68.0%, and 92.5%	200 adults (feces), 200 poultry (ceca/feces), 120 wastewater	[8]
Burkina Faso	67.5%	202 *E. coli* isolates (clinical)	[9]
Cameroon	34.4%	90 *E. coli* isolated from blood culture of children (clinical)	[10]
Côte d'Ivoire	27%, 32%, 0%	77 people, 38 dogs, 75 wildlife	[11]
Democratic Republic of the Congo (DRC)	98.2%	57 multidrug-resistant *E. coli* isolates (clinical)	[12]
Ethiopia	17.4%	224 *E. coli* isolates (clinical)	[13]
Kenya	44%	406 children (clinical)	[14]
Mali	22%	136 *E. coli* isolates (clinical)	[15]
Mozambique	32.6%	230 clinical	[16]
Nigeria	32.2%	115 *E. coli* isolates (poultry workers, chickens, farm/market environments)	[17]
Senegal	36.7%	49 slaughterhouse effluent	[18]
Tanzania	21.7%	350 children (rectal) (clinical)	[19]
Uganda	50%	42 multidrug-resistant *E. coli* isolates from stool (30 people, 12 cattle)	[20]

**Table 2:** Collaborating countries receiving GHSA support on AMR containment through the program

• Bangladesh • Burkina Faso • Cameroon • Côte d'Ivoire	• DRC • Ethiopia	• Kenya • Mali • Mozambique • Nigeria	• Senegal • Tanzania • Uganda

A number of factors contribute to less effective AMR control in LMICs, such as poorer sanitation and hygiene [21]; limited access to quality antimicrobials, diagnostics, and vaccines [22]; and more inappropriate use of antibiotics—overuse, underuse, and misuse, among others [23, 24]. Poor infection prevention and control (IPC) practices, which include hand hygiene in health facilities and water, sanitation, and hygiene (WASH) issues, have a major effect on AMR. Less-resourced countries also have a high prevalence of health care-associated infections (average 12.8% in Africa) [25], and IPC awareness and systems are generally poor in both the human and animal sectors [26]. Barriers to improve usage and awareness through antimicrobial stewardship (AMS) in low-income countries include lax regulations and weak enforcement of existing regulations regarding antimicrobial availability and use in humans and animals [27], limitations in the laboratory capacity to detect drug-resistant microorganisms [28], and lack of clinician training in these areas. However, a multinational survey revealed a 114% increase in antibiotic consumption in LMICs between 2000 and 2015 [29], and a study showed that between 2007 and 2017, children in 8 LMICs received, on average, 25 antibiotic prescriptions from birth through 5 years, which is up to 5 times higher than the already high levels observed in high-income settings [30]. Rising antibiotic consumption in LMICs combined with the extensiveness of their AMR drivers amplifies the already grave situation in these countries and the world.

As of November 2022, 170 World Health Organization (WHO) member states had finalized their national action plans on AMR (NAP-AMR) [31], which mostly align with the approaches laid out in the 2015 WHO Global Action Plan [32]. AMR cannot be overcome without addressing all of its drivers spanning the human, animal, plant, and environmental sectors, so the One Health approach [33, 34], with multisectoral coordination (MSC) at its core, provides the mechanisms for countries to successfully implement their multisectoral NAP-AMR, including strengthening IPC and AMS practices in human and animal health. However, LMICs struggle with funding and operationalizing these plans—nearly 40% lack a budgeted operational plan [35]. One major reason is that the NAP tends to be a plan of plans [36], with several strategic objectives and a long list of recommended actions under each objective; this poses challenges on where to start and on what to focus relative to LMICs’ capacities and available funding.

Since September 2018, the mandate of the US Agency for International Development’s Medicines, Technologies, and Pharmaceutical Services (MTaPS) program (henceforth called “the program”) has been to work with 13 countries (Table 2) to make Global Health Security Agenda (GHSA)-supported progress on the implementation of their NAP-AMR in the specific areas of MSC, IPC, and AMS; US Agency for International Development designated the countries to receive program support. Partners in these efforts comprise government counterparts in the human and animal sectors and other in-country stakeholders such as health facilities, health professional associations, civil society, nongovernmental organizations, academia, and the private sector including the pharmaceutical industry. On the global front, collaborations include donors and their implementing partners; UN bodies such as the WHO, the Food and Agriculture Organization, and the World Organisation for Animal Health; as well as the US Centers for Disease Control and Prevention. Based on our multicountry experience, this paper discusses four promising practices for strengthening countries’ AMR containment capacity.

The program bases its technical approach on the second edition of the Joint External Evaluation (JEE) tool (2018) [37] and WHO Benchmarks for International Health Regulations (IHR) Capacities (2019) [38], which help countries achieve their goals under the GHSA. The JEE tool [37]—a key component of the monitoring and evaluation framework for IHR [39]—is used to evaluate the IHR capacity requirements, including national AMR containment capacity, which is one of the GHSA’s 19 technical areas. The GHSA initiative’s aim is to raise countries’ capacities incrementally from no capacity (level 1) to sustainable capacity (level 5). As of May 2022, 116 countries had completed the JEE [40]. Analyses of JEE scores have shown that AMR remains one of the weakest technical areas [41–43], highlighting the need for continued support to increase countries’ AMR containment capacity.

Table 3 shows the baseline JEE scores in IPC and AMS for the program’s 13 GHSA partner countries. Overall, countries had more capacity in IPC than in AMS. Four countries, however, were evaluated as having no capacity (1) in either area, while Uganda’s scores were the highest—developed capacity (3) in IPC and AMS. The first edition of the JEE tool did not include the multisectoral coordination on AMR (MSC-AMR) indicator, so the table does not reflect baseline scores for that area. The second version, released in 2018, does include an MSC-AMR indicator as does the 2019 WHO benchmarks tool; therefore, we used those documents to assess countries’ status and guide implementation in MSC-AMR. In addition, the countries’ baseline JEE reports included recommendations that applied to MSC. For example, Bangladesh’s 2016 report included this recommendation, “The National Action Plan should be updated to align with the Global Action Plan for antimicrobial resistance and then finalized. Steps should be taken to implement the plan and indicators developed to follow progress” [44].

**Table 3:** Baseline JEE scores for 13 target countries in IPC and AMS using 2016 JEE tool

Country	JEE date	P.3.3 IPC	P.3.4 AMS
Bangladesh	May 2016	2	2
Burkina Faso	December 2017	1	1
Cameroon	September 2017	1	1
Côte d'Ivoire	December 2016	1	1
DRC	March 2018	1	1
Ethiopia	March 2016	2	2
Kenya	February–March 2017	3	2
Mali	June 2017	2	1
Mozambique	April 2016	3	1
Nigeria	June 2017	2	2
Senegal	November–December 2016	3	1
Tanzania	February 2016 (mainland)	3	1
	April 2017 (Zanzibar)	1	1
Uganda	June 2017	3	3

The JEEs provide valuable baseline status of countries’ national capacities in various technical areas but moving from evaluations to capacity-strengthening actions has been difficult. The 2019 WHO benchmarks tool complements the JEE tool and provides an organized framework with capacity-level appropriate actions for incremental progress in the JEE technical areas. Our program is one of the first mechanisms to use the tool as a principal guide in its GHSA support for AMR containment.

We made scoping visits to 11 of the 13 collaborating countries. Our program in Mozambique and Nigeria started in October 2020 during the height of the COVID-19 pandemic, so the initial information on these two countries was based mainly on document reviews and long-distance consultations. Our scoping exercises determined that most collaborating countries had finalized their NAP-AMR, but their operationalization was weak, and many had not drafted costed implementation plans. Mechanisms for multisectoral coordination on AMR existed in some form, but countries struggled with functionality and needed help with establishing and/or strengthening their technical working groups (such as those for IPC and AMS). In IPC, policies and guidelines mostly existed but needed support with updating, implementing, and monitoring. Most countries lacked antimicrobial consumption and use data as well as AMS policies, guidelines, and programs, which required elementary support; for example, several countries, including Mali and DRC, had no oversight structure for drug and therapeutics committees, and although Côte d’Ivoire had a collection of AMS guidelines, there was no one national policy or set of guidelines.

**Fig. 1:**
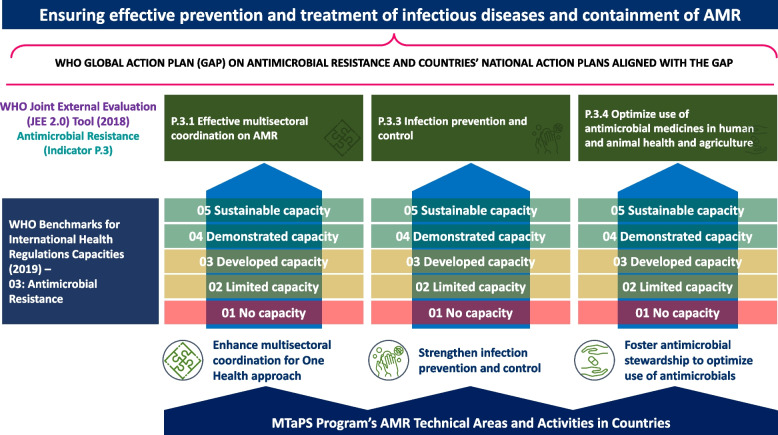
Program’s GHSA-supported technical approach to align with national and global goals for AMR containment

We supported capacity improvement in the 13 countries in 3 of the 4 AMR containment indicators included in the 2018 version of the JEE tool—MSC on AMR (P.3.1); IPC (P.3.3); and optimizing the use of antimicrobial medicines in human and animal health and agriculture through AMS (P.3.4) (Fig. 1).

## Methods

The program’s implementation plans were based on country scoping visits, baseline JEE scores, and national- and facility-level assessments to identify gaps and strengths and inform priorities that align with the actions recommended in the WHO benchmarks tool. Based on these results, we identified common needs in areas such as leadership and enabling environment, local capacity strengthening, and monitoring/self-learning, which have been the focus of our technical assistance since our work with countries began.

We build on countries’ existing structures, such as multisectoral mechanisms on AMR and coordinate with partners to leverage their resources. The program applies diverse methods to strengthen local capacity and works closely with in-country stakeholders in the transfer of technologies (such as monitoring and data-sharing platforms, standard treatment guidelines app, and WhatsApp groups) and the transfer of competencies through training-of-trainers and cascade training and onsite skills-building support; creating centers of excellence; and improving the capacity of national, subnational, and facility governance entities, professional associations, and civil society organizations. We strategically collaborate with government counterparts and other implementing partners in the self-propagation and scale-up of evidence-based tools, techniques, and approaches.

The program fosters vertical coordination between national- and facility-level stakeholders and horizontal coordination between facilities through peer mentorship and cross-fertilization. Supportive supervision and mentoring are a collaborative effort among national stakeholders and program staff using pre-agreed checklists. The aim of this support is to strengthen the local capacity to achieve and maintain best practices in IPC and AMS for the long term.

Listed below are the key activities the program supports in the three technical areas.

### Strengthening multisectoral collaboration through stakeholder engagement

- Strengthening leadership and governance functions or technical capacity of the multisectoral (One Health) coordination body on AMR [45]
- Helping to set up or improve the functioning of national technical working groups on IPC and AMS
- Supporting the development and update of IPC, AMS, and MSC governance documents
- Facilitating collaboration between the human and animal health sectors and seeking opportunities to also engage the environmental sector
- Helping to revise countries’ NAP-AMR and develop a monitoring and evaluation framework and operational/implementation plan for countries’ NAP-AMR

### Growing infection prevention and control programs and improving practices

- Evaluating IPC programs and practices in facilities using the WHO IPC assessment framework (IPCAF) [46] and national-level IPC programs using the WHO IPC assessment tool version 2 (IPCAT2) [47] to inform actions and monitor progress on WHO IPC core components [48]
- Conducting more specific facility assessments of compliance with IPC guidelines by using the WHO hand hygiene self-assessment framework and adapted WHO IPC scorecards
- Supporting facility-level health care-associated infection point prevalence surveys and the design and implementation of health care-associated infection surveillance
- Helping to develop or update IPC guidelines, standard operating procedures, and action plans at the national and facility levels
- Helping to establish and make functional national IPC committees or technical working groups and facility IPC committees
- Incorporating quality improvement approaches for facilities to identify, address, and monitor IPC issues
- Building capacity in IPC competencies in facility staff and other categories of health care personnel through training, eLearning, mentoring, and supportive supervision

### Improving awareness of antimicrobial stewardship and addressing deficits

- Conducting rapid assessments of country laws, regulations, and guidelines related to AMS and antimicrobial supply chains
- Conducting facility AMS assessments to inform actions based on WHO-recommended AMS core elements [49]
- Capacitating government counterparts to use WHO standardized methods and tools, such as the Anatomical Therapeutic Chemical (ATC) and defined daily doses (DDD) methodology and point-prevalence survey [50], to conduct surveillance of antimicrobial consumption and use, respectively
- Incorporating the WHO AWaRe (access, watch, reserve) classification of antibiotics [51, 52] in pharmaceutical governance documents such as the national essential medicines lists, standard treatment guidelines, and formularies
- Promoting the creation and function of national AMS committees or technical working groups
- Supporting the development of AMS documents such as policies, guidelines, and plans
- Supporting the establishment and strengthening of facility drug and therapeutics committees to provide AMS-related oversight
- Developing facility AMS action plans and helping to determine priority areas
- Strengthening AMS training and including practical AMS topics in pre-service curricula and in-service training

As indicated, WHO developed most of the tools used. Some were adapted in partnership with national stakeholders for local country contexts and needs before implementation. For example, several countries adapted IPCAT2 or IPCAF tools to assess IPC programs related to animal health and agriculture, and in Bangladesh, COVID-19 elements were incorporated into standardized IPC assessments. To ensure quality results in the assessments, country stakeholders met to review and validate the drafts before finalizing.

A GHSA 2024 Framework strategic objective [53] is to improve the sharing of best practices and lessons regionally and globally. Toward this goal, the program has focused on gleaning promising practices from its implementation experiences and lessons (as the results section below describes) and sharing them to advance the GHSA-related regional and global learning agenda.

## Results

Since 2018, our work with the 13 countries, guided by the WHO JEE and benchmarks tools, points to four promising practices for countries to strengthen AMR containment (summarized in Table 4).

**Table 4:** Strengthening AMR Containment in LMICs: Four Promising Practices Highlighted through the Program’s 13-country Experience

1. Implement capacity level-appropriate actions using the WHO Benchmarks for IHR Capacities as an organizing framework
• The JEE tool is used to assess country capacity in containing AMR; however, it is the complementary WHO benchmarks tool that offers practical guidance to prioritize specific capacity-appropriate actions that can incrementally augment AMR-related capacities
• Although the WHO benchmarks tool is primarily designed to use with JEEs to support GHSA and IHR, it provides an organized framework based on existing country capacity levels, and thus can be used more broadly to simplify and prioritize NAP implementation actions. In addition, standardized tools and methods such as IPCAT2, IPCAF, WHO point prevalence survey tool, and AWaRe classification of antimicrobials are used to assess specific areas and focus on the benchmark actions that improve IPC and AMS programs and practices
2. Identify entry points and integrate AMR into other national and global agendas
• To raise its profile as a national priority, AMR should be promoted as a health security threat that is an essential element of pandemic preparedness
• Given that AMR has the potential to affect all aspects of clinical care and public health relating to infectious diseases, AMR containment efforts should be mainstreamed into broader agendas such as universal health coverage (UHC) and other national and subnational programs, such as quality improvement (QI)/quality of care (QoC), WASH, and maternal and child health; however, practical examples of countries operationalizing this approach are lacking. If this approach can be successfully scaled up, it can help break the traditional siloed approaches and also address the chronic lack of funding for AMR containment in LMICs
3. Improve governance through multisectoral coordination on AMR
• The second (2018) and third (2022) editions of the JEE emphasize the importance of MSC in the AMR technical area. Reinforcing governance such as organizational management in MSC bodies and providing policy support in areas including funding improve their function and ability to coordinate AMR containment efforts across sectors. Strengthening MSC governance at subnational and local level is equally important, but stewardship needs to stay at the central level
• MSC bodies and their technical working groups, if functioning effectively, can catalyze both advocacy and actions against AMR in the spirit of One Health. However, attention should be paid to make sure that MSC efforts go beyond just meetings and lead to practical joint actions. Therefore, all relevant ministries and stakeholders should be included in planning and implementing activities to ensure that they coordinate with each other; typically, the human health sector works more actively in AMR containment, while other sectors need targeted engagement
• MSC bodies and their technical working groups are well-suited to forge integrative collaboration with non-traditional stakeholders such as those working in climate change, which now is being recognized as an AMR threat multiplier
4. Mobilize and diversify funding for AMR containment efforts
• Regular mapping of players and programs in various sectors can help identify opportunities for collaboration and funding for AMR-related actions. Additionally, integrating AMR and NAP activities into government program agendas and budgets helps diversify funding, resulting in more sustainable resources that are not donor-based
• MSC bodies must advocate for support from in-country decision- and policy-makers and politicians who are crucial for securing longer-term funding. Preparing a costed operational plan/investment case for AMR containment provides evidence to strengthen this advocacy

### Implement capacity level-appropriate actions using the WHO benchmarks for IHR capacities as an organizing framework

Although JEEs provide clear national capacity milestones to reach level 5, they provide little guidance on which actions to choose from an extensive inventory, which is a barrier to countries’ progress. The WHO benchmarks tool addresses this critical gap by recommending and prioritizing actions aligned with the JEE tools’ capacity framework, making it easier for countries to incrementally increase JEE capacity from level 1 to 5. Table 5 shows that the 13 countries have achieved notable progress on benchmark actions in MSC, IPC, and AMS from September 2018 to September 2022 with the program’s support. For example, Côte d'Ivoire scored level 1 for IPC and AMS in their December 2016 baseline JEE assessment; by September 2022, it had completed 100% (5/5) of level 2 benchmark actions in IPC; 100% (6/6) of level 3 actions; and 80% (4/5) of level 4 actions. In AMS, Côte d'Ivoire also completed 75% (3/4) of level 2 benchmark actions, 83% (5/6) of level 3 actions, and 29% (2/7) of level 4 actions. Although MSC has no JEE baseline scores due to its absence in the 2016 edition of the JEE tool, the program helped Côte d’Ivoire achieve robust progress in this area by supporting 100% (4/4) of level 2 benchmark actions; 75% (3/4) of level 3 actions; and 75% (3/4) of level 4. Other countries made similar headway on benchmark actions along the path to higher capacity levels (Table 5). However, some benchmark actions have only been partially met because they include two or more components within the same action (e.g., both human and animal sector components).

**Table 5:** WHO benchmarks tool (2019) actions fully or partially^a^ supported by MTaPS as of September 2022^b^

**Joint External Evaluation (JEE) capacity level**^c^	**Country**^d^												
	**Bangladesh**	**Burkina Faso**	**Cameroon**	**Côte d'Ivoire**	**DRC**	**Ethiopia**	**Kenya**	**Mali**	**Mozambique**	**Nigeria**	**Senegal**	**Tanzania (mainland)**	**Uganda**
**P.3.1 Effective multisectoral coordination on AMR**^e^													
*JEE Capacity Baseline Score*	No baseline scores, as the first version of the JEE tool (2016) used for these evaluations did not have indicator on effective multisectoral coordination on AMR												
Limited Capacity—02 (4 actions)	0%	50%	50%	100%	50%	100%	50%	0%	50%	0%	50%	0%	50%
Developed Capacity—03 (4 actions)	25%	50%	50%	75%	50%	100%	50%	75%	50%	50%	50%	50%	50%
Demonstrated Capacity – 04 (4 actions)	75%	50%	25%	75%	75%	100%	100%	100%	25%	75%	50%	75%	50%
Sustainable Capacity—05 (5 actions)	20%	0%	0%	0%	20%	0%	40%	20%	0%	20%	20%	0%	0%
**P.3.3 Infection prevention and control**													
*JEE Capacity Baseline Score*^g^ *(Month and year of JEE)*	2 (May 2016)	1 (Dec 2017)	1 (Sep 2017)	1 (Dec 2016)	1 (Mar 2018)	2 (Mar 2016)	3 Feb-Mar 2017)	2 (Jun 2017)	3 (Apr 2016)	2 (Jun 2017)	3 (Nov-Dec 2016)	3 (Feb 2016)	3 (Jun 2017)
Limited Capacity—02 (5 actions)	80%	N/A^f^	80%	100%	80%	80%	80%	100%	80%	60%	60%	80%	100%
Developed Capacity—03 (6 actions)	83%	N/A	83%	100%	67%	100%	67%	83%	67%	67%	100%	100%	100%
Demonstrated Capacity – 04 (5 actions)	20%	N/A	60%	80%	0%	80%	60%	40%	0%	0%	60%	100%	40%
Sustainable Capacity—05 (5 actions)	0%	N/A	0%	0%	0%	0%	0%	0%	0%	0%	40%	100%	0%
**P.3.4 Optimize use of antimicrobial medicines in human and animal health and agriculture**													
*JEE Capacity Baseline Score*^g^	2	1	1	1	1	2	2	1	1	2	1	1	3
Limited Capacity—02 (4 actions)	50%	50%	50%	75%	75%	75%	75%	75%	50%	100%	75%	100%	50%
Developed Capacity—03 (6 actions)	33%	17%	33%	83%	33%	67%	83%	50%	50%	50%	33%	50%	33%
Demonstrated Capacity – 04 (7 actions)	0%	0%	29%	29%	0%	14%	14%	14%	14%	0%	0%	14%	29%
Sustainable Capacity—05 (7 actions)	0%	0%	0%	0%	0%	0%	0%	0%	0%	0%	0%	0%	0%
**Total number of MTaPS-supported actions (%)**	**19/62 (31%)**	**9/41 (22%)**^f^	**23/62 (37%)**	**33/62 (53%)**	**21/62 (34%)**	**34/62 (55%)**	**30/62 (48%)**	**27/62 (44%)**	**19/62 (31%)**	**20/62 (32%)**	**26/62 (42%)**	**33/62 (53%)**	**25/62 (40%)**

^a^Some benchmark actions were partially achieved as they comprise two or more separate components, some of which MTaPS did not support

^b^This table reflects only those WHO benchmark actions that MTaPS supported, so is not a comprehensive picture of the country’s AMR containment capacity. Support for some benchmark actions was ongoing as of September 2022

^c^There are no benchmark actions associated with level 01 (no capacity)

^d^MTaPS initiated GHSA support in October 2020 in Mozambique and Nigeria, December 2019 in Bangladesh, and between September 2018 and March 2019 in the remaining countries. MTaPS did not have activities in Ethiopia from December 2020 to September 2021

^e^The JEE baseline was conducted using the first (2016) edition of the tool, which unlike the second (2018) edition, did not include indicator for ‘effective multisectoral coordination on AMR’

^f^MTaPS does not support IPC activities in Burkina Faso

^g^Some of the actions that MTaPS supported corresponded to the actions recommended for the same level as the JEE baseline score levels or even below the baseline levels, because we found that they had not been addressed yet

The WHO benchmarks tool also serves as a yardstick for in-country stakeholders to periodically self-assess advancement toward the next level. For example, Cameroon, Côte d’Ivoire, DRC, and Nigeria used the benchmarks tool to internally measure improvement and prioritize next actions. This approach helps stakeholders better prepare for the annual Tripartite AMR country self-assessment survey (TrACSS) process [54] and recurring JEEs, which are recommended every four to five years [39].

The IPC benchmark actions emphasize repeat IPC assessments to measure incremental progress due to iterative interventions [38]. WHO provides several tools to assess IPC programs, including IPCAT2 for the national level and IPCAF for the facility level. As of September 2021, our facility counterparts in 12 countries had carried out baseline IPCAF assessments^1^ in the program-supported facilities and repeated assessments in 71 public and private sector facilities in 9 countries. Of those facilities, the scores in 37 (52%) had increased by at least 1 capacity level. Table 6 illustrates how helping the IPC committees use a QI approach to help implement the national IPC guidelines, WHO multimodal IPC strategy, and IPC activities resulted in substantial increases in IPC capacity scores in five Senegal hospitals, with two hospitals jumping by two of four capacity levels. Recognition of IPC’s cross-cutting importance has elevated it from being just an AMR indicator in JEE 2 to a separate technical area in the new JEE 3 [40].

**Table 6:** IPCAF review of program-supported hospitals in Senegal before and after IPC improvement actions

Hospitals	IPCAF baseline score/800 and capacity level February–March 2021	IPCAF follow-up score/800 and capacity level October 2021
Level 1 Mbour Hospital	167.5 Inadequate	455 Intermediate
Level 2 Fatick Hospital	315 Basic	513 Intermediate
Level 2 Kaffrine Hospital	380 Basic	535 Intermediate
Level 3 Touba Hospital	310 Basic	450 Intermediate
Level 3 Aristide le Dantec Hospital	322 Basic	692.5 Advanced

The WHO benchmarks tool recommends actions around monitoring antimicrobial consumption and/or use in each of the capacity levels 2 to 5. Without access to reliable data on AMR and antimicrobial consumption and use, it is difficult to establish and run an AMS program and to carry out other AMS benchmark actions to increase JEE capacity. It is therefore critical to build the capacity of government counterparts to conduct and interpret studies that characterize national antimicrobial consumption and/or facility antimicrobial use as in the case of DRC, Tanzania, and Uganda (Table 7). At the national level, the studies involved working with the public and private sector including local manufacturers and suppliers with results allowing for performance comparisons to other countries. Public health experts in these countries now have the data to identify and implement evidence-based improvement interventions and the skills to institutionalize the methods to mark decreases in inappropriate national- and facility-level antimicrobial consumption and use. In addition to strengthening country capacity to carry out antimicrobial consumption and use surveillance, by publishing their results in peer-reviewed journals, we also helped enhance counterparts’ skills in how to draft and publish scientific articles.

**Table 7:** Studies conducted to generate reliable data on antimicrobial consumption and use

**DRC:** National consumption study covering 2018–2019 [55, 56]
**Collaborators**: Directorate of Pharmacy and Medicine and other national stakeholders, WHO, MTaPS program **Tool**: WHO ATC/DDD methodology **Setting**: Retrospective consumption study for January 2018 to December 2019 with Kinshasa, Haut Katanga, and Nord-Kivu as the data collection sites **Findings**: • 85% of the antimicrobials were used in the private sector, 13% were used with development partners’ support, and only 2% were used in the public sector • Aggregate consumption increased from 12 to 16 DDD per 1,000 persons/day from 2018 to 2019 • Approximately 70% of antibiotics consumed were in the ‘access’ group of AWaRe categories
**Tanzania**: National Consumption of Antimicrobials in Tanzania: 2017–2019 [57]
**Collaborators**: Tanzanian Ministry of Health, Tanzania Medicine & Medical Devices Authority, St. John’s University of Tanzania, University of Washington, MTaPS program **Tool**: WHO ATC/DDD methodology **Sample**: Data on all antimicrobials imported into Tanzania (2017–2019), purchasing data from the Medical Stores Department, and data from local manufacturers **Findings**: • The DDD per 1,000 population per day declined from 136.41 in 2017 to 54.98 in 2018 and 51.02 in 2019 • Most antimicrobial consumption occurred in the private sector, with the proportion increasing annually from 2017 to 2019 • > 90% of antimicrobial consumption was ‘access’ medications, with ‘watch’ and ‘reserve’ medications accounting for < 10% and < 1%, respectively
**Tanzania:** Antimicrobial use across six referral hospitals in Tanzania: a point prevalence survey [58] **Collaborators:** Tanzanian Ministry of Health, Catholic University of Health and Allied Sciences, University of Washington, MTaPS program **Tool**: WHO point prevalence tool **Sample and setting**: 948 patients from 6 referral hospitals **Findings**: • Approximately 62.3% of inpatients were prescribed antibiotics. Children less than 2 years of age, admission to surgical and pediatric wards, and being male were associated with increased odds of being prescribed antibiotics • Prescriptions were predominantly from the AWaRe ‘access’ group of antibiotics with an average of 84.0% in compliance with the standard treatment guidelines • Only 2 of 591 patients were prescribed antibiotics based on culture and antimicrobial susceptibility testing results
**Uganda**: Point Prevalence Survey of Antibiotic Use across 13 Hospitals in Uganda [59]
**Collaborators**: Ministry of Health, Makerere University School of Health Sciences, University of Washington, Overseas Strategic Consulting, Ltd., MTaPS program **Tool**: WHO point prevalence tool **Sample and setting**: 1,077 patients from 13 hospitals nationwide **Findings**: • 74% of patients were on at least one antibiotic. Males were more likely to be prescribed an antibiotic compared to females, and public hospitals were significantly more likely to be associated with antibiotic use than private hospitals • Compliance with the Uganda Clinical Guidelines was low (30% of antibiotics prescribed) • A high proportion of prescriptions (44%) included antibiotics from the WHO ‘watch’ classification, primarily due to the high use of ceftriaxone, which was prescribed most frequently • Very high use of parenteral antibiotics (88%) compared to oral use (12%)

The WHO benchmarks tool recommends integrating AWaRe categories of antibiotics into national governance documents such as national essential medicines lists and standard treatment guidelines. DRC, Kenya, Mali, and Tanzania incorporated AWaRe categories in such documents using a checklist the program developed [60]. By institutionalizing WHO’s AWaRe classification of antibiotics, countries can incorporate it into their metrics and use the results to inform the design of evidence-based AMS activities. As of March 2022, two of these four countries had mandated using AWaRe for antibiotic selection in all health facilities; one country’s national health insurance fund added AWaRe as a reimbursement requirement; three countries had conducted antibiotics consumption/use surveys that included AWaRe; and one country’s national regulatory authority had issued AWaRe-related regulatory guidance to health professionals. In addition, because the JEE includes regulatory indicators on medicine use in the human and animal health sectors, the program created a standardized method to rapidly assess AMS-related policies, laws, regulations, and practices in both sectors. As of September 2022, seven countries had used the method to assess the situation in both the human and animal sectors and inform AMS priorities, leading to post-assessment actions such as development of national AMS plans in Cameroon, Côte d’Ivoire, Mali, and Senegal, and drafting of a ministerial order regulating AMS in the animal sector in Burkina Faso. Since the findings of these IPC and AMS assessment tools inform the design of interventions that address weaknesses, they help national stakeholders and implementing partners institutionalize the tools for improvement, monitoring, and evaluation.

Institutionalizing the monitoring of IPC and AMS practices is an important benchmark to achieve JEE level 5—sustainable capacity. The Uganda Ministry of Health instituted a standardized IPC supportive supervision checklist, while the Senegal Ministry of Health integrated IPC into its standard supportive supervision tool and staff training. The ministry of health in Tanzania incorporated AMS into its nationwide Afya Supportive Supervision program and added IPC indicators to those collected in the DHIS2, thus ensuring that facilities routinely collect and report IPC data as part of a systems-strengthening approach. The Uganda Ministry of Health sent a circular to health facilities’ leadership that approved and recommended appropriate medicine use and monitoring activities, including point prevalence surveys to support AMS.

### Identify entry points and integrate AMR into other national and global agendas

Ongoing agendas and programs at international, regional, and national levels provide opportunities to mainstream and interlink AMR containment efforts [36]. Several potential entry points exist, including initiatives in QI/QoC, WASH, and maternal and child health. In Tanzania for example, three hospitals acted immediately, especially on things that did not require resources, after we conducted WASH assessments and staff orientation on WASH and IPC guidelines and assessment results. The hospitals installed elbow-driven handwashing sinks, repurposed unused equipment, and recruited an implementing partner to help install a water storage tank. Countries are also increasingly applying QI/QoC approaches in health service delivery [61]. In Uganda, for example, we are using GHSA support to enhance QI/QoC approaches by linking WASH, HIV, and maternal and child health to improve AMR containment and creating hospital centers of excellence to improve IPC and AMS service quality and mentor other facilities. QI/QoC has similarly proven to be an effective entry point in Bangladesh, Senegal, and Tanzania. NAPs typically cover five years, and as countries update them, they can map such linkages and mention them more explicitly; for example, although WASH and AMR containment are highly interrelated, we noted during the initial period of the program’s support that only one of the 13 collaborating countries mentioned WASH as an integrated terminology and practice area in their NAP-AMR.

### Improve governance through multisectoral coordination on AMR

We helped to establish or guide IPC and AMS technical working groups in 9 countries and AMR secretariats in 11 countries to prioritize, plan, and review activities. Through this work, we found that reinforcing governance in MSC bodies and their technical working groups—for example, by improving terms of reference, membership diversity, vertical coordination, and meeting frequency—enhances their functionality [45]. We worked with MSC bodies to include members from the private sector, professional associations, and civil society on their national committees and working groups, while advocating for better gender balance in MSC meetings and actions.

The human health sector in our 13 collaborating countries tends to be more active in AMR containment compared to the animal health, fisheries, or environment sectors, and MSC meetings are typically led by the ministry of health. However, our work with MSC bodies and working groups using the GHSA lens has improved animal sector engagement, providing impetus for involving the environmental sector. In Mozambique, for example, we brokered consensus between human health and animal health representatives to rotate responsibilities for running the MSC mechanism on AMR and its technical working groups. From a practical standpoint, inspired by the WHO IPC assessment tools for the human health sector, and without similar tools for IPC assessment in the animal sector, Côte d’Ivoire, DRC, and Mali adapted IPCAT2 and/or IPCAF to conduct IPC assessments in the animal sector. Mali conducted its first national-level IPC assessment in the animal health sector using a modified IPCAT2, which led to the development of IPC guidelines and an action plan. A hygiene and IPC rapid assessment was done in 10 veterinary practices, 8 slaughterhouses, and 33 poultry farms in Côte d’Ivoire using tools adapted from IPCAF. Burkina Faso developed its first national guidelines for using antibiotics in the animal sector, and in Uganda, the program coordinated the development of the first veterinary essential medicines list and five guidelines on infection prevention and use of antimicrobials in food-producing animals. Coordination between national counterparts in both the human and animal sectors produced these achievements.

COVID-19 has highlighted how systems-based and institutionalized MSC and IPC initiatives can strengthen pandemic preparedness and contribute to rapid responses. Our prior work to strengthen governance in MSC and IPC helped national stakeholders respond more quickly to COVID-19 in several ways [45]. For example, MSC bodies or their members expedited response coordination, and IPC working groups were able to quickly turn their attention to the pandemic. IPC guidance documents and training efforts were easily adapted; in several countries, IPC eLearning had already been initiated prior to the pandemic, and countries such as Côte d’Ivoire, Cameroon, and Kenya already had IPC master trainers who incorporated COVID-19 into their teaching. In addition, in Kenya, we collaborated with representatives of seven health professional associations^2^ to develop continuing professional development and re-licensure-linked AMS and IPC courses that the associations have taken over and deliver online to their members who work in both the public and private sectors; in just over two months from October to December 2020, during the height of the pandemic, more than 1,000 health association members completed virtual sessions on AMS and earned credit from regulatory bodies that contributed to their qualification to renew their annual practice licenses. Similar initiatives in Ethiopia and Côte d’Ivoire working with civil society and professional associations proved effective in expanding training and collaboration among stakeholders, including those from the private sector.

### Mobilize and diversify funding for AMR containment efforts

Many of the countries that we support rely primarily on donor funding for their national AMR program, and the MSC bodies and their technical working groups tend to be inadequately funded. Major outbreaks often cause a temporary surge in political commitment and budget allocations, but countries generally struggle with advancing and sustaining their JEE capacities because of insufficient resources and competing national priorities [62]. Therefore, the program is collaborating with MSC bodies and their IPC and AMS working groups in several partner countries to map and advocate for funding for AMR-related activities. For example, in Ethiopia, Cameroon, and Senegal, MSC bodies drafted and submitted proposals to the Tripartite AMR Multi-Partner Trust Fund, a funding vehicle for multisectoral coordination on NAP implementation [63]; Ethiopia’s and Senegal’s proposals were successfully funded. Côte d’Ivoire, Ethiopia, and Tanzania used WHO’s resource mapping and impact analysis on health security investment (REMAP) tool to map and assemble financial and technical assistance resources to carry out national health security plans [64]. Repeat REMAPs in Côte d’Ivoire have shown visible implementation progress in the AMR technical area—from 5% in 2019 to 40% in 2020, 53% in 2021, and 70% by June 2022—all as a result of stronger MSC and funding mobilization.

## Discussion

AMR is an invisible threat, which impedes its ascendence as a national priority. GHSA and IHR provide a health security perspective by framing the issue in terms of outbreak prevention and preparedness. This perspective can complement national AMR containment efforts, and it is important to integrate AMR with other national-level GHSA technical areas. Fields such as immunization, zoonotic diseases, and food safety are integral for reducing the burden of infectious diseases and antibiotic consumption. As such, national AMR programs and immunization programs should work closely with each other to promote proven vaccines to reduce infections and need for antibiotics [65]. Robust examples, preferably from LMIC settings, of how countries integrate AMR into other initiatives and activities are urgently needed to help translate and roll out this valuable concept as a standard approach. Equally critical is multisectoral collaboration to quickly develop and deploy vaccines to address infectious disease outbreaks and also to help decrease inappropriate use of antibiotics; COVID-19 provides a stark example of how antibiotics can be overused and misused during a pandemic [66], which amplifies the risk of AMR.

Linking AMR containment to global and national health priorities such as UHC and the Sustainable Development Goals can leverage synergies [36]; in fact, two new AMR indicators were added to the SDG monitoring framework in 2020 [67]. Because infections remain a significant cause of disease and death in LMICs, UHC efforts will not be sustainable without conserving antimicrobial effectiveness and ensuring the quality and safety of care through strong IPC and WASH practices. Most elements to attain UHC and contain AMR are mutually reinforcing [68]; for example, integrating AWaRe classification of antibiotics into essential medicines lists—a level 3 benchmark action—expands the list’s use from a tool that primarily supports procurement and supply chain management to one that also supports AMS; a simple process like this contributes to essential health services by supporting both access and rational use. MSC, IPC, AMS, and surveillance, when implemented with a focus on systems-strengthening, lead to a ‘triple win’ of achieving the objectives of AMR containment, pandemic preparedness, and UHC.

GHSA, JEE, and IHR have substantially advanced MSC, which has been hailed as a paradigm shift toward One Health [69]. The 2nd edition of the JEE tool (2018) elevated MSC-AMR by adding a dedicated indicator (P.3.1.). The newly released 3rd edition of the JEE tool (2022) [40] has continued to emphasize MSC as a critical indicator for the AMR technical area. Many countries now have national-level MSC [70], but efforts are needed to further enable MSC bodies to go beyond advocacy and joint meetings to catalyze actions that can serve as solid examples of One Health at the operational level [71]. MSC bodies and their technical working groups should liaise with professional associations, civil society organizations, private sector, and other organizations aiming to advance One Health. With the growing global momentum for One Health and Planetary Health, the MSC-AMR platforms also have opportunities to expand coalitions beyond the traditional AMR players, for example collaborating with groups working in climate change, as it is now being recognized as a potential AMR threat multiplier [72]. In addition, MSC activities must occur not just at the national level but also at subnational levels, which Ethiopia, Kenya, and Nigeria have initiated. Building decentralized and localized multisectoral and multidisciplinary capacity has many advantages including proximity, faster implementation, access to local resources, and local ownership. However, central-level stewardship must continue. For efficient functioning, institutionalization, and sustainability, MSC bodies require enabling policy support including those related to human resources, capacity-building, and funding [45].

While donor funding can be vital in initiating AMR-related activities, the WHO benchmarks tool highlights the integral role of financing with recommended actions related to funding mobilization and diversification [38], and sustainability will depend on long-term funding commitments from government and other domestic resources [36]. Targeting both large and small funding sources can lead to diversified funding streams. Among others, the private sector can be a key partner in funding activities against AMR; for instance, Pfizer and Biomerieux pledged support for the MSC bodies’ AMR activities in Côte d'Ivoire and Uganda. In addition, strategically integrating AMR into relevant national and departmental agendas and programs can also help yield and diversify funding. The level 5 benchmark action in MSC (P.3.1)—Ensure key activities are incorporated in plans and budgets of relevant programs and agencies—points to that approach’s importance. Having a costed operational plan for priority activities [73, 74] provides evidence to advocate for funding, and investment cases with clear political and economic arguments for integrating the NAP into annual budgets and for mobilizing funds are necessary to sustain progress [75]. It is therefore critical to frequently update the mapping of initiatives and stakeholders that could be approached for leveraging collaboration, including funding, for specific aspects of the prioritized and costed plans.

While the 2019 WHO benchmarks tool provided a useful set of recommended capacity-appropriate actions based on JEE 2, we also noted some limitations while helping implement the tool in the collaborating countries. A key observation was that several AMR-related actions had two or more components requiring different streams of efforts and stakeholders combined within a single action, such as including both animal and human health-related work in AMS and IPC areas and developing AMS plans and legislation. Additionally, some of the IPC actions recommend the use of several tools in the same action, making it hard to demonstrate progress based on partial or selected use of the tools. Importantly, while the tool recommends human and animal sector actions, it does not mention any specific environmental sector-related actions within the AMR technical area. Also, neither JEE 2 nor the 2019 benchmarks tool links IPC actions to pandemic preparedness capacity. JEE 3 has addressed some of these limitations and now separates human and animal actions for AMS and mentions the role of IPC in outbreaks and pandemics.

## Conclusions

WHO has declared that AMR is one of the top 10 public health threats the world faces [1]. AMR must be addressed not only as a threat to individuals and communities but also to national and global health security—the devastating COVID-19 pandemic has shown the criticality of investments in health security and pandemic preparedness. There is growing recognition that AMR has the potential to become a similar pandemic without concerted global actions. If we are to make any impact in containing AMR, LMICs must increase their abilities to strengthen their health systems and programs needed to implement their NAP-AMR in multiple sectors and at multiple levels. Experience from our multicountry collaborations over several years indicates improved animal sector involvement, but continued focus is needed to further consolidate their engagement. The environmental sector has been minimally included, and urgent efforts are needed to identify entry points for their participation in LMICs. Additionally, countries need innovative strategies to make private sector, civil society, media, and the community major allies and partners. LMICs need additional focus on strengthening their regulatory systems, going beyond the traditional educational and managerial types of AMR containment interventions. Similarly, our experience from Kenya shows that incorporating national, county, facility, and community levels truly diversifies and localizes AMR containment actions.

Country stakeholders and partners must move beyond just assessments and recommendations and start implementing locally feasible AMR containment actions. Our experience shows that regular, locally led follow-up is needed after initial assessments to prioritize and implement interventions, as are repeat assessments using the same tools to demonstrate evidence-based improvements, such as those seen through repeated IPCAT2 and IPCAF assessments in several of our partner countries. The WHO benchmarks tool is valuable to help countries prioritize such actions based on existing capacities to incrementally improve their JEE capacity through multisectoral governance and other efforts. Although the tool is primarily designed for use in conjunction with JEEs to support GHSA and IHR, it could potentially be used more broadly to simplify and prioritize NAP-AMR implementation actions.

The path to sustaining best IPC, AMS, and surveillance practices and multisectoral coordination under One Health requires a health systems strengthening approach that builds on locally existing systemic foundations and capacities; lasting transfer of skills in the use of proven tools, technologies, and methodologies; and country ownership and institutionalization of initiatives. In this manner, national stakeholders, donors, and partners will continue to help countries move toward achieving demonstrated (level 4) capacity or a comparable level in several technical areas in the coming years, thereby contributing to GHSA [76].

In this paper, we presented four promising practices highlighted through our 13-country collaboration experience and discussed country examples that could potentially be scaled. As LMICs continue to tackle the growing threat of AMR using the WHO benchmarks and other tools, sharing lessons learned and best practices and highlighting critical gaps will continually improve the technical resources available to strengthen their AMR containment capacity.

## Data Availability

Not applicable.

## Abbreviations

AMR: Antimicrobial resistance
AMS: Antimicrobial stewardship
ATC: Anatomical Therapeutic Chemical classification system
AWaRe: Access, watch, reserve
DDD: Defined daily dose
DRC: Democratic Republic of the Congo
DHIS2: District Health Information System version 2
ESBL: Extended-spectrum β-lactamase
GHSA: Global Health Security Agenda
IHR: International Health Regulations
IPC: Infection prevention and control
IPCAF: IPC assessment framework (facility level)
IPCAT2: IPC assessment tool version 2 (national level)
JEE: Joint External Evaluation
LMICs: Low- and middle-income countries
MSC: Multisectoral coordination
MTaPS: Medicines, Technologies, and Pharmaceutical Services
NAP-AMR: National action plans on AMR
QI: Quality improvement
QoC: Quality of care
TrACSS: Tripartite AMR country self-assessment survey
UHC: Universal health coverage
WASH: Water, sanitation, and hygiene
WHO: World Health Organization

## Footnotes

1 Kenya used Infection Control Assessment Tool (ICAT), not IPCAF.

2 National Nurses Association of Kenya, Kenya Clinical Officer Associations, Association of Kenya Medical Laboratory Scientific Officers, Kenya Society for Physiotherapists, Kenya Pharmaceuticals Association, Kenya Medical Association, and the Pharmaceutical Society of Kenya.
